# The Corporeality of Late Age

**DOI:** 10.1093/geroni/igab046.2265

**Published:** 2021-12-17

**Authors:** Chris Gilleard

**Affiliations:** University College London, UCL, England, United Kingdom

## Abstract

In a study of over a thousand Germans, Paul Baltes and his colleagues observed that most respondents saw age 80-84 as the preferred age to reach before dying. Living beyond 85 was only desired by a minority. Perhaps this is because this age seems to many the point when bodily disease and physical weakness render life not just unpleasant but actively burdensome. Such views underpin the social imaginary of an undesirable fourth age. This paper discusses the significance of corporeality as both representation and lived experience, raising the question of whether the disparity between real and imagined corporealities resides as much from an ‘other’ within as without.

